# Proposal for a Domain Wall Nano-Oscillator driven by Non-uniform Spin Currents

**DOI:** 10.1038/srep14647

**Published:** 2015-09-30

**Authors:** Sanchar Sharma, Bhaskaran Muralidharan, Ashwin Tulapurkar

**Affiliations:** 1 Department of Electrical Engineering, Indian Institute of Technology Bombay, Powai, Mumbai 400076, India

## Abstract

We propose a new mechanism and a related device concept for a robust, magnetic field tunable radio-frequency (rf) oscillator using the self oscillation of a magnetic domain wall subject to a uniform static magnetic field and a spatially non-uniform vertical dc spin current. The self oscillation of the domain wall is created as it translates periodically between two unstable positions, one being in the region where both the dc spin current and the magnetic field are present, and the other, being where only the magnetic field is present. The vertical dc spin current pushes it away from one unstable position while the magnetic field pushes it away from the other. We show that such oscillations are stable under noise and can exhibit a quality factor of over 1000. A domain wall under dynamic translation, not only being a source for rich physics, is also a promising candidate for advancements in nanoelectronics with the actively researched racetrack memory architecture, digital and analog switching paradigms as candidate examples. Devising a stable rf oscillator using a domain wall is hence another step towards the realization of an all domain wall logic scheme.

Self oscillators show a remarkable property of sustaining oscillatory behavior without being driven by sources that possess inherent periodicity. In the macroscopic world, a few well known examples of self oscillations include the heartbeat, violin string oscillations in response to steady bowing^1^, the Vander Pol oscillator and the infamous collapse of the Tacoma Narrows Bridge in 1940^2^. In the nanoscale too, self oscillations govern the underlying principle of the well studied resonant tunneling diode based oscillator, the spin torque oscillator^3^,^4^,^5^ and also, the recently noted phenomenon of nuclear spin induced current oscillations in quantum dots^6^. Our proposal relies on the self oscillations in the translatory motion of a magnetic domain wall.

The interest in dynamics of magnetic domain walls has been active for decades^7^,^8^,^9^,^10^,^11^,^12^, recently intensified by the discovery of current-driven domain wall motion^13^,^14^,^15^ and its related applications in nanoelectronics^5^,^16^,^17^,^18^. Field driven oscillations have also been observed and studied^11^,^19^ for a long time. However, these oscillations are accompanied by a drift which makes them unusable as a device. On a different note, vertical injection of uniform spin current is proposed as a means for high domain wall velocities^20^,^21^,^22^. Here, we propose stable oscillations caused by a constant magnetic field whose drift is canceled by a vertically injected non-uniform spin current as depicted in the schematic in Fig. 1(a), thereby resulting in a stable periodic motion.

Normally, domain wall motion under vertical spin currents is caused by *field like torque* which is typically smaller than the *Slonczewski like torque*^22^,^23^,^24^,^25^ usually responsible for the switching of the free layer of magnetic tunnel junctions (MTJ)^26^. However, the presence of a magnetic field allows an efficient transfer of the Slonczewski like torque without any need of a field like term, as shown later. We note that current-driven domain wall oscillations with a drift have been observed^27^ while stable oscillations on a pinned domain wall were proposed^28^. Our proposal differs from the latter in the control of oscillations via an external field instead of a pinning potential, both of which under rigid domain wall approximation work to rotate the domain wall.

We first provide a theoretical analysis using a rigid domain wall model to find an approximate waveform for the oscillations. We show that the frequency is twice the resonant frequency of a magnet in a magnetic field; while the amplitude is approximately a linear function of the ratio of the hard axis anisotropy and the magnetic field. The oscillatory part of the waveform is independent of the input spin current to a very good degree of accuracy; and hence can be of great technological advantage for accurate oscillatory waveforms. We then also numerically simulate the micromagnetic Landau-Lifshitz-Gilbert equation (see supplementary material) as a verification of the oscillations and find the frequency to be in good agreement with the analytic result. Lastly, we analyze the effect of thermal noise on the oscillator and find that it is robust at room temperature by numerically calculating its Q-factor.

The working principle of the proposed oscillator is depicted in Fig. 1(a). The vertically incident spin current is spatially confined to the left half of the domain wall, while the uniform magnetic field along the −*z* direction exists throughout the region. Both the regions, 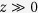 and 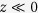 by themselves are unstable for the domain wall (unless the spin current is too low) and hence it is restricted to be in some region around *z* = 0. A possible device realization of the proposal, in which the domain wall self-oscillations may be effectively translated into an alternating current oscillations is depicted in Fig. 1(b). A dc-current source is connected to the bottom strip of Tantalum, which is used to inject a spin current via the giant spin hall effect (SHE)^29^,^30^,^31^. The strip of Tantalum conveniently acts as a source of spin current that injects a constant spin current in a locally confined region marked in the schematic in Fig. 1(a). An MTJ like structure is then used to sense the position of the domain wall via a measurement of the change in resistance^20^. A small current is applied and the corresponding voltage across the MTJ is measured. An equivalent circuit for the entire set up is shown in Fig. 1(c) depicting the measurement in a more explicit way. We assume that the current used for the measurement is small enough such that it doesn’t have any additional effect on the domain wall dynamics.

The analysis to follow will be based on Fig. 1(a) which captures the essence of our proposal. The region of non-zero spin current “pushes” the domain wall towards the region of zero spin current via spin transfer torque (STT). However, the domain wall cannot keep moving away from the spin current as the magnetic field will push it back via the dissipative Gilbert term^9^. As shown in Fig. 1(d), when the domain wall enters the spin current region, it is reflected back with a different azimuthal angle accumulated because of the magnetic field. The hard axis anisotropy then keeps the domain wall moving until the magnetic field rotates the domain wall again to cause reverse motion. Hence, the magnetic field causes a perpetual rotation, while the hard axis anisotropy converts the rotation into a translation of the domain wall. The spin current then acts as the energy input which negates the dissipative effect present in a purely field driven motion, hence stopping the drift observed in the latter case. For a typical case of a low dissipation constant, the domain wall will need only a small amount of push from the spin current and hence the oscillations will be almost independent of it. The average location of the domain wall though will be dependent on the spin current density. Our analysis is based on the magnetization dynamics of the domain wall described by the Landau-Lifshitz-Gilbert equation augmented by the Slonczewski spin torque term^23^ given by


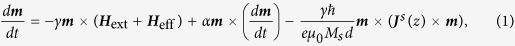


where ***m***(*z*, *t*) is the magnetization unit vector; *γ*(>0) is the gyromagnetic ratio; *M*_*s*_ is the saturation magnetization of the magnet; *μ*_0_ is the permeability of free space; *d* is the thickness of the sample (Fig. 1(b)); *α* is the Gilbert dissipation constant; ***J***^*s*^(*z*) is the vertical spin current density loss which is assumed to be dependent only on *z*; ***H***_ext_ is the externally applied field; 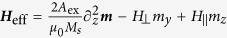; *A*_ex_ is the exchange energy constant; 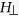 is the hard axis anisotropy; 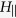 is the easy axis anisotropy. The above equation can be derived from the Lagrangian along with the generalized forces (see supplementary information) given by the following expressions,


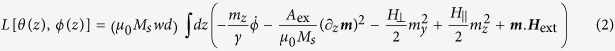



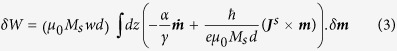


where *w* is the width of the magnet (see Fig. 1(b)). We consider the rigid domain wall ansatz^32^, 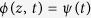 and 
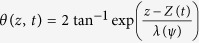
, where the “width” of the domain wall, 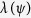, is given by 
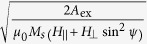
. We consider the case of a spin current polarized only along the ‘*z*’ axis with its expression being 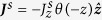 where *θ* is the Heaviside function. Additionally we have a uniform magnetic field along −*z* direction, 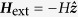. Finally, we get the equation of motion as,


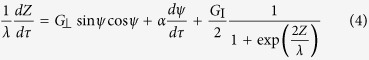



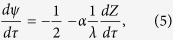


where the dimensionless time is defined as 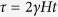 and we define constants 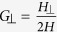 and 
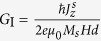
. The frequency can directly be deduced from eqs, (4) and (5), as follows. Let there exist an oscillatory solution of *Z* and *λ* with a common dimensionless time (*τ*) period, say T. Note that under such conditions, all the terms in Eq (4) are periodic with period T except possibly the term containing 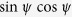. For this term to be periodic with T, 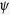 has to change by an integer multiple of *π* after the time period. Considering Eq (5), it can be seen that 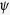 has an oscillatory term along with a drift of “speed” −1/2. Using the known value of “speed” of 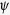, we can conclude that any oscillations have to exist with a dimensionless time period of 2*nπ* where *n* is any natural number. We restrict our attention to T = 2*π* which is what we observe in simulation. Coming back to real time, *t*, we conclude that rigid domain wall approximation restricts the angular frequency of oscillation to be 2*γH* (see Eq (7)). Note that with this time period, we indeed have an oscillation in *λ* with the same dimensionless time period.

If *G*_I_ = 0, the equation for 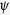 can be solved exactly^9^ and *Z* can be integrated using Eq (5). With 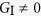, there does not seem to be an analytic solution. However, using the intuition that spin current is a small perturbation which mainly acts to negate the effect of dissipation, we can use the solution for field driven motion^9^ to approximate the oscillations as,


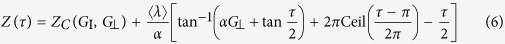


where Ceil is the ceiling function; *Z*_*C*_ is a constant dependent on spin current and fields while the oscillatory part is dependent only on 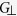; 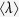 is the average value of *λ* which can be approximated as 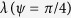. We can now use Eq (6) to analyze the waveform explicitly to calculate various useful observables. First, we use it to find the amplitude of oscillation as 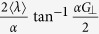. We also note that for 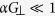, this expression reduces approximately to Eq (8). The fact that hard axis anisotropy is responsible for converting rotation into motion is backed up by the expression of the amplitude of the oscillation being approximately proportional to the ratio of the hard axis anisotropy and the magnetic field (see eq 8). To calculate the threshold of spin current, we take the time average of Eq (4). Then using the waveform (see Eq (6)) in Eq (5), we get the inequality, 
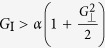
. This is in accordance with the intuition that the spin current mainly acts to negate the effect of dissipation present in the field driven motion of a domain wall. The results after dismantling the notation are summarised in Eq (7), (8) and (9).


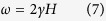



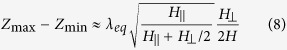



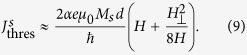


where *λ*_*eq*_ is the equilibrium value of *λ* and the factor in the square root (Eq (8)) arises due to the variation in the width of the domain wall under oscillatory motion. It will be absent if the variation in width is neglected or in other words, 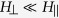.

We now demonstrate the simulated results of the domain wall motion using the rigid wall approximation discussed above. The waveform derived in Eq. (6) is not an exact solution of the equations for the rigid domain wall, but matches fairly well with the numerics as shown in Fig. 2(a). The regime of operation of the device as shown in Fig. 2(b) demonstrates that we need a minimum magnitude of the spin current to compete against the magnetic field and hence result in the oscillations. This can be understood by analyzing the motion of the domain wall when it starts from deep inside either region, i.e., the region deep inside the region of zero or non-zero spin current.

In Fig. 3(a), we demonstrate the simulated motion of a rigid domain wall starting from a point *z* < 0. As shown in the figure, the domain wall will be pushed away until the “force” of the spin current is small enough to be compensated by the drift caused by the magnetic field. An opposite scenario is shown in Fig. 3(b), where the domain wall starting deep inside the region of zero spin current (*z* > 0) will have a field driven drift until it encounters the region of non-zero spin current. In both the scenarios, the spin current magnitude should be large enough to push back the domain wall or the latter will continue to move indefinitely against the field.

To verify the results, we have also performed micromagnetic simulations, details of which are included in the supplementary material. We consider a 3 nm thick magnetic film with a cross-section of 800 × 100 nm. We assume the magnet parameters, 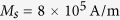 and 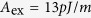; where *M*_*S*_ is the saturation magnetization and *A*_ex_ is the exchange energy. We assume a crystalline anisotropy, with its contribution to energy density as 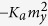 where 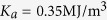, in the direction of its thickness which works toward reducing the hard axis anisotropy caused by the dipolar interaction. We apply a magnetic field of 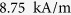 and a spin current density of 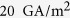. We have simulated it for 40 ns to find that oscillations occur at a frequency close to 0.56 GHz. This value is close to the one derived using the theoretical analysis as written in Eq (7).

## Noise Analysis

**Figure 4:**
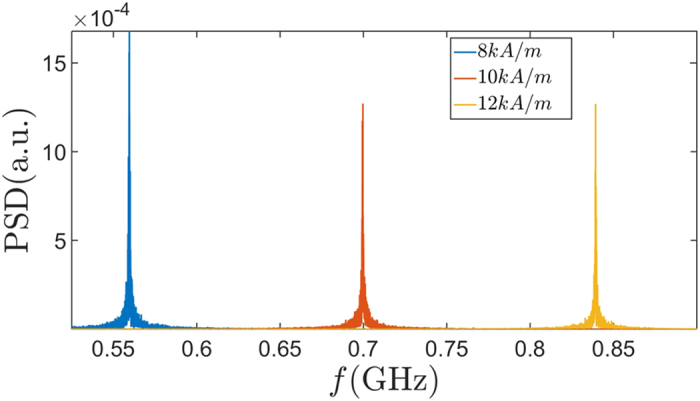
Noise Analysis. Power Spectral Density of the domain wall position for three values of the applied field and with the spin current density fixed at 0.96 *GA*/*m*^2^. It shows peaks at the expected values of the oscillation frequency with small width due to noise.

Finally, we verify the stability of the oscillations by adding noise to the rigid domain wall equations and numerically calculating its quality factor. For simplicity, we assume 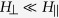 which corresponds to a domain wall with constant width. We introduce two uncorrelated white noise sources, *N*_*Z*_ and 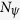, both of which satisfy 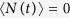 and 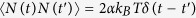. The noise can then be added as^33^,


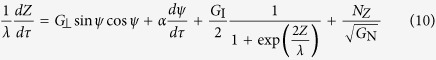



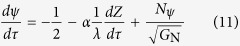


where 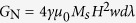 has been chosen such that Fokker-Planck equation corresponding to Langevin equations Eq (10) and Eq (11) admits the Boltzmann distribution in steady state. We simulated the above equations at a room temperature of 300 *K* for 40 *μs* for various values of spin current and magnetic field. The power spectral density (PSD) of *Z* for three values of the applied magnetic field is plotted in Fig. 4. From the simulated spectrum we find that the quality factor of the oscillator is ~550, ~1100 and ~1400 respectively for the applied fields of 8 *kA*/*m*, 10 *kA*/*m* and 12 *kA*/*m* respectively.

In conclusion, we have proposed a new set up for an oscillator based on the self oscillations of a magnetic domain wall. We found that under rigid domain wall approximation, the oscillatory part of waveform is almost independent of input spin current and the frequency of oscillations is solely governed by the external magnetic field. We also demonstrated a high quality factor giving evidence for the stability of the oscillations. We envision that the simple set up proposed, namely a domain wall subject to a non-uniform vertical spin current will also open up many possibilities for simultaneous write and read out along with the possibility of an all domain wall logic scheme.

## Additional Information

**How to cite this article**: Sharma, S. *et al.* Proposal for a Domain Wall Nano-Oscillator driven by Non-uniform Spin Currents. *Sci. Rep.* **5**, 14647; doi: 10.1038/srep14647 (2015).

## Supplementary Material

Supplementary Information

Supplementary Information 1

## Figures and Tables

**Figure 1:**
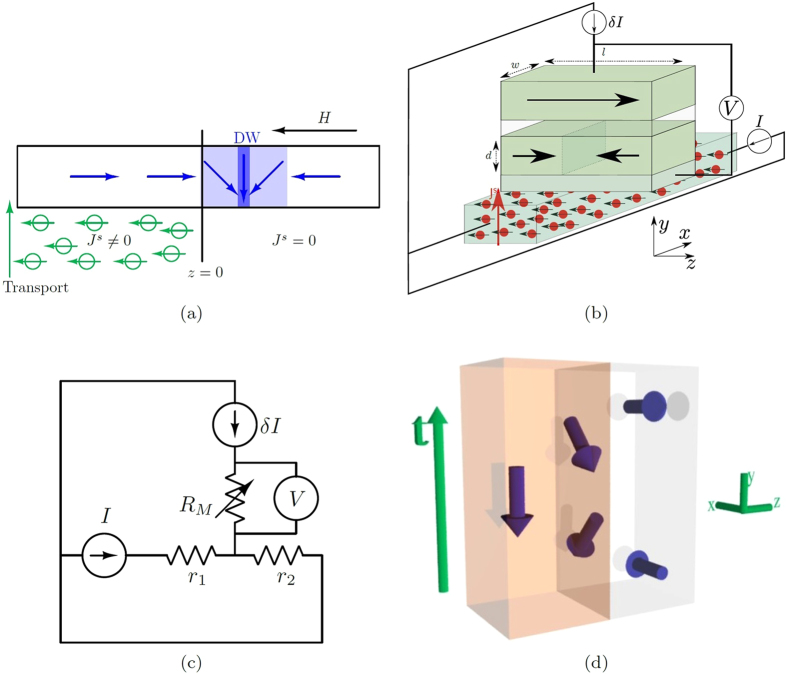
Working principle and device design. (**a**) Schematic depicting the principal idea of the proposal which comprises a domain wall magnet with the incident non-uniform spin current and a uniform magnetic field. The domain wall is shown at an arbitrary instantaneous position. The non-uniform spin current is incident on a region spanning one-half of the magnet. (**b**) A 3D schematic of the proposed device design. A strip of Tantalum localized in the desired region of the domain wall magnet, connected to a current source generates the desired spin current. The applied magnetic field along the −*z* axis is not shown here for clarity. (**c**) A circuit diagram representing the device schematic in (**b**). *r*_1_ is the resistance of the part of Tantalum strip between the constant current source, ‘I’, and MTJ, and similarly *r*_2_. (**d**) Zoomed-in motion of middle spin marked in (**a**), depicting the rotation it undergoes which gets converted into motion via its hard axis anisotropy. The orange colored region is the portion where spin current is non-zero.

**Figure 2:**
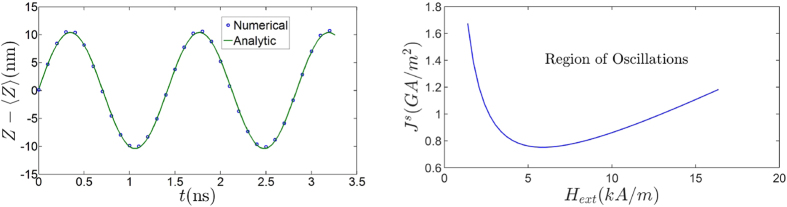
Oscillatory waveform and region of occurence (**a**) Comparison of the oscillatory part of the numerical and the analytic solution of domain wall position vs time, under an external applied field of 10 *kA*/*m*. The spin current density for the numerical solution was taken to be 0.96 *GA*/*m*^2^. (**b**) The values of spin current and magnetic field depicting the region in which the oscillations happen.

**Figure 3:**
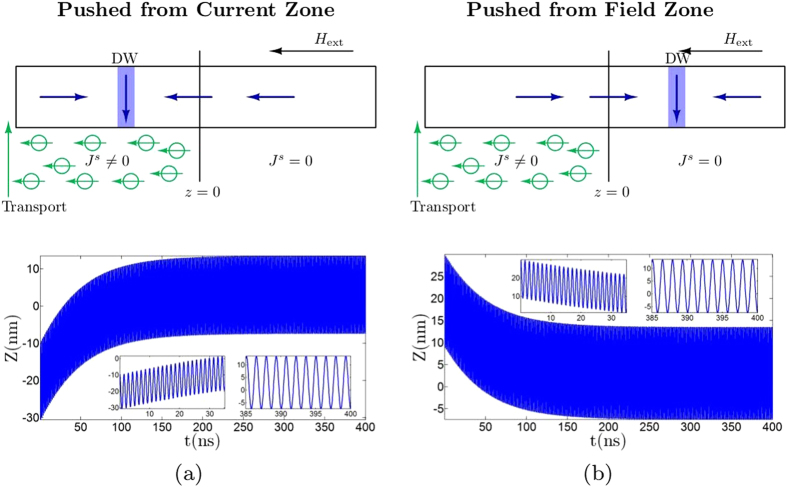
Current push and Field push. (**a**) Simulation results for domain wall position, *Z* when started from a point inside the region of non-zero spin current. (**b**) Similar plot as (**a**) except for the initial position of domain wall being inside the region of zero spin current.
