# Supplementary material for: Proposal for a Domain Wall Nano-Oscillator driven by Non-uniform Spin Currents
Source: Sci Rep. 2015 Sep 30;5:14647. doi: 10.1038/srep14647 (PMC4588506; doi:10.1038/srep14647)
Supplement: Supplementary Information [file srep14647-s1.pdf]

Supplementary Information - Proposal for a Domain Wall Nano-Oscillator driven by Non-uniform Spin Currents

Sanchar Sharma[1], Bhaskaran Muralidharan[1,*], and Ashwin Tulapurkar[1]

[1]Department of Electrical Engineering, Indian Institute of Technology Bombay, Mumbai, 400076, India
[*]bm@ee.iitb.ac.in

ABSTRACT

Here, we provide details about the rigid wall approximation along with the details of the micromagnetic simulations performed in the paper.

1 Derivation of Lagrangian

The starting point of magnetization dynamics is the Landau-Lifshitz-Gilbert (LLG) equation augmented with Slonczweski spin torque[(1)] term

$$\frac{d\boldsymbol{m}}{dt} = -\gamma \boldsymbol{m} \times \boldsymbol{H}_{\text{eff}} + \alpha \boldsymbol{m} \times \left(\frac{d\boldsymbol{m}}{dt}\right) - \frac{\gamma\hbar}{e\mu_0 M_s d} \boldsymbol{m} \times (\boldsymbol{J}^s(z) \times \boldsymbol{m}) \tag{1}$$

where $\boldsymbol{J}^s(z)$ is the incident spin current density which is assumed to be dependent only on z; $\boldsymbol{H}_{\text{eff}} = \dfrac{-1}{\mu_0 M_s} \dfrac{\delta \tilde{E}}{\delta \boldsymbol{m}}$ where $\tilde{E}$ is the micromagnetic energy density. eq (1) can be derived from the Lagrangian, eq (2), along with the generalized forces derived from the expression in eq (3),

$$L = \mu_0 M_s w d \int dz \left(-\frac{m_z}{\gamma} \dot{\phi} - E[\boldsymbol{m}](z) \right) \tag{2}$$

$$\delta W = \mu_0 M_s w d \int dz \left(-\frac{\alpha}{\gamma} \dot{\boldsymbol{m}} + \frac{\hbar}{e\mu_0 M_s d} (\boldsymbol{J}^s \times \boldsymbol{m}) \right) . \delta \boldsymbol{m} \tag{3}$$

where $\mu_0 M_s E = \tilde{E}$. In this section, we derive this equivalence. We take $\{m_z(z), \phi(z)\}$ as coordinates. Hence, we can write,

$$\delta \boldsymbol{m} = \sin\theta \, \delta\phi \, \hat{\boldsymbol{\Phi}} + \delta\theta \, \hat{\boldsymbol{\Theta}} \tag{4}$$

where $\cos\theta = m_z$, $\hat{\boldsymbol{\Phi}} = (-\sin\phi, \cos\phi, 0)$ and $\hat{\boldsymbol{\Theta}} = (\cos\theta\cos\phi, \cos\theta\sin\phi, -\sin\theta)$. This gives $\dot{\boldsymbol{m}}.\delta\boldsymbol{m} = \sin^2\theta \dot{\phi} \delta\phi + \dot{\theta}\delta\theta$ which translates to,

$$\dot{\boldsymbol{m}}.\delta\boldsymbol{m} = \sin^2\theta \dot{\phi}\delta\phi + \frac{\dot{m}_z \delta m_z}{1 - m_z^2} \tag{5}$$

One can notice that $\{\boldsymbol{m}, \hat{\boldsymbol{\Theta}}, \hat{\boldsymbol{\Phi}}\}$ forms an orthonormal right handed system. They are just the unit vectors in polar coordinates with $\boldsymbol{m}$ acting like the radius vector. Using Euler Lagrange equations by varying the action with respect to the coordinates, we get two equations of motion,

$$\frac{d}{dt}\frac{\delta L}{\delta \dot{\phi}} - \frac{\delta L}{\delta \phi} = \frac{\delta W}{\delta \phi} \Rightarrow$$

$$-\dot{m}_z + \gamma \frac{\delta E}{\delta \phi} = -\alpha(1 - m_z^2)\dot{\phi} - \frac{\gamma\hbar}{e\mu_0 M_s d} \sqrt{1 - m_z^2} \boldsymbol{J}^s . \hat{\boldsymbol{\Theta}} \tag{6}$$

$$\frac{d}{dt}\frac{\delta L}{\delta \dot{m}_z} - \frac{\delta L}{\delta m_z} = \frac{\delta W}{\delta m_z} \Rightarrow$$

$$\dot{\phi} + \gamma \frac{\delta E}{\delta m_z} = -\alpha \frac{\dot{m}_z}{1 - m_z^2} - \frac{\gamma\hbar}{e\mu_0 M_s d} \frac{\boldsymbol{J}^s . \hat{\boldsymbol{\Phi}}}{\sqrt{1 - m_z^2}} \tag{7}$$

The above equations are equivalent to the Landau-Lifshitz Gilbert (LLG) equation. To show this, we explicitly reduce down the terms in eq (6) to relevant terms in eq (1).

$$\gamma\frac{\delta E}{\delta\phi} = \gamma\frac{\delta E}{\delta m_y}\sin\theta\cos\phi - \gamma\frac{\delta E}{\delta m_x}\sin\theta\sin\phi \tag{8}$$

$$= -\gamma(\boldsymbol{m}\times\boldsymbol{H}_{\text{eff}})_z \tag{9}$$

$$\alpha(1-m_z^2)\dot{\phi} = \alpha(1-m_z^2)\frac{m_x\dot{m}_y - m_y\dot{m}_x}{m_x^2+m_y^2} \tag{10}$$

$$= \alpha\left(\boldsymbol{m}\times\dot{\boldsymbol{m}}\right)_z \tag{11}$$

$$\sqrt{1-m_z^2}\boldsymbol{J}^s.\hat{\boldsymbol{\Theta}} = \sin\theta\boldsymbol{J}^s.\left(\boldsymbol{m}\times\hat{\boldsymbol{\Phi}}\right) \tag{12}$$

$$= \boldsymbol{J}^s.\left(\boldsymbol{m}\times(\hat{\boldsymbol{z}}\times\boldsymbol{m})\right) \tag{13}$$

$$= -\left(\boldsymbol{m}\times(\boldsymbol{J}^s\times\boldsymbol{m})\right)_z \tag{14}$$

From the above set of equalities, we can readily write eq (6) as,

$$\frac{d\boldsymbol{m}_z}{dt} = -\gamma(\boldsymbol{m}\times\boldsymbol{H}_{\text{eff}})_z + \alpha\left(\boldsymbol{m}\times\frac{d\boldsymbol{m}}{dt}\right)_z - \frac{\gamma\hbar}{e\mu_0 M_s d}\left(\boldsymbol{m}\times(\boldsymbol{J}^s(z)\times\boldsymbol{m})\right)_z \tag{15}$$

which is just the z-component of eq (1). Hence, one of the equations we get from Lagrangian is compatible with LLG. For the next equation, eq (7), we proceed from eq (1) and show that we can derive eq (7),

$$\hat{\boldsymbol{\Phi}}.\dot{\boldsymbol{m}} = -\gamma\hat{\boldsymbol{\Phi}}.(\boldsymbol{m}\times\boldsymbol{H}_{\text{eff}}) + \alpha\hat{\boldsymbol{\Phi}}.(\boldsymbol{m}\times\dot{\boldsymbol{m}}) - \frac{\gamma\hbar}{e\mu_0 M_s d}\boldsymbol{J}^s.\hat{\boldsymbol{\Phi}} \Rightarrow$$

$$\sin\theta\dot{\phi} = -\gamma\hat{\boldsymbol{\Theta}}.\boldsymbol{H}_{\text{eff}} + \alpha\hat{\boldsymbol{\Theta}}.\dot{\boldsymbol{m}} - \frac{\gamma\hbar}{e\mu_0 M_s d}\boldsymbol{J}^s.\hat{\boldsymbol{\Phi}} \Rightarrow$$

$$\sin\theta\dot{\phi} = \gamma\left[H_{\text{eff},z}\sin\theta - \cos\theta\left(H_{\text{eff},x}\cos\phi + H_{\text{eff},y}\sin\phi\right)\right] - \alpha\frac{\dot{m}_z}{\sin\theta} - \frac{\gamma\hbar}{e\mu_0 M_s d}\boldsymbol{J}^s.\hat{\boldsymbol{\Phi}} \Rightarrow$$

$$\dot{\phi} = \gamma\left[H_{\text{eff},z} - \frac{m_z}{1-m_z^2}\left(H_{\text{eff},x}\cos\phi + H_{\text{eff},y}\sin\phi\right)\right] - \alpha\frac{\dot{m}_z}{1-m_z^2} - \frac{\gamma\hbar}{e\mu_0 M_s d}\frac{\boldsymbol{J}^s.\hat{\boldsymbol{\Phi}}}{\sqrt{1-m_z^2}} \Rightarrow$$

$$\dot{\phi} = -\gamma\frac{\delta E}{\delta m_z} - \alpha\frac{\dot{m}_z}{1-m_z^2} - \frac{\gamma\hbar}{e\mu_0 M_s d}\frac{\boldsymbol{J}^s.\hat{\boldsymbol{\Phi}}}{\sqrt{1-m_z^2}} \tag{16}$$

Hence, we show that both eq (6) and eq (7) are satisfied if the original LLG equation eq (1) is. As the number of independent equations in LLG is 2, we can say that the former two equations are equivalent to LLG.

2 Derivation of the Rigid Domain Wall Approximation

We concluded the last section by deriving a Lagrangian for the magnetization dynamics. In this section, we use rigid domain wall approximation to derive the equation of motion for the collective coordinates. This amounts to putting the following ansatz in the Lagrangian,

$$\phi(z,t) = \psi(t) \tag{17}$$

$$\theta(z,t) = 2\tan^{-1}\left(e^{\frac{z-Z(t)}{\lambda(t)}}\right) \tag{18}$$

After we put this ansatz in the Lagrangian eq (2), we can carry out the integration with respect to the z co-ordinate. The energy functional (divided by $\mu_0 M_s$) is,

$$E = \frac{A_{\text{ex}}}{\mu_0 M_s}(\partial_z\boldsymbol{m})^2 + \frac{H_\perp}{2}m_y^2 - \frac{H_\parallel}{2}m_z^2 - \boldsymbol{m}.\boldsymbol{H}_{\text{ext}} \tag{19}$$

Then, we will be left with a Lagrangian with Z, ψ and λ as coordinates. It is this final Lagrangian that will give us the equation of motion via the Euler-Lagrange equations. The generalized force due to STT, however, will have to be changed as well. By the prescription of work-energy, we have

$$\int dz\left(\boldsymbol{J}^s\times\boldsymbol{m}\right).\delta\boldsymbol{m} = F_\psi\delta\psi + F_Z\delta Z + F_\lambda\delta\lambda \tag{20}$$

where $\{F_\psi, F_Z, F_\lambda\}$ are functions of $\{Z, \psi, \lambda\}$ which we are going to find. For convenience in notation, we resolve $\boldsymbol{J}^s$ as,

$$\boldsymbol{J}^s(z) = J_1(z)\boldsymbol{m}(z) + J_2(z)\boldsymbol{m}'(z) + J_3(z)\boldsymbol{m}(z)\times\boldsymbol{m}'(z) \tag{21}$$

where $\boldsymbol{m}'(z)$ denotes the derivative of $\boldsymbol{m}(z)$ w.r.t z. The above expansion is valid only if $\boldsymbol{m}'$ is non-zero everywhere. This is true in the particular ansatz we are considering. We write down the relevant expressions now,

$$\boldsymbol{m}'.(\boldsymbol{m}\times\delta\boldsymbol{m}) = -\sin^2\theta\frac{\delta\psi}{\lambda} \tag{22}$$

$$\boldsymbol{m}'.\delta\boldsymbol{m} = -\sin^2\theta\left(\frac{\delta Z}{\lambda^2} + \frac{\delta\lambda(z-Z)}{\lambda^3}\right) \tag{23}$$

Now, using the above equations, the term under consideration is (refer (21) for definition of J_i),

$$\boldsymbol{J}^s.(\boldsymbol{m}\times\delta\boldsymbol{m}) = J_2\boldsymbol{m}'.(\boldsymbol{m}\times\delta\boldsymbol{m}) + J_3\boldsymbol{m}'.\delta\boldsymbol{m} \tag{24}$$

$$= -J_2\theta'\sin\theta\delta\psi + J_3\theta'\delta\theta \tag{25}$$

$$= -\sin^2(\theta(z))\left[J_2(z)\frac{\delta\psi(t)}{\lambda(t)} + J_3(z)\left(\frac{\delta Z}{\lambda^2} + \frac{\delta\lambda(z-Z)}{\lambda^3}\right)\right] \tag{26}$$

$$\int dz\boldsymbol{J}^s.(\boldsymbol{m}\times\delta\boldsymbol{m}) = F_\psi\delta\psi + F_Z\delta Z + F_\lambda\delta\lambda \tag{27}$$

$$F_\psi = -\int\frac{dz}{\lambda}\frac{4}{\left[\exp\left(\frac{z-Z}{\lambda}\right) + \exp\left(-\frac{z-Z}{\lambda}\right)\right]^2}J_2(z) \tag{28}$$

$$F_Z = -\int\frac{dz}{\lambda^2}\frac{4}{\left[\exp\left(\frac{z-Z}{\lambda}\right) + \exp\left(-\frac{z-Z}{\lambda}\right)\right]^2}J_3(z) \tag{29}$$

$$F_\lambda = -\int\frac{dz}{\lambda^3}\frac{4(z-Z)}{\left[\exp\left(\frac{z-Z}{\lambda}\right) + \exp\left(-\frac{z-Z}{\lambda}\right)\right]^2}J_3(z) \tag{30}$$

So far, it was generally applicable for any distribution of spin currents. For the case under consideration, we focus on a specific spin current distribution such that J^z is a constant, say J^s_z, for $z < 0$ and zero for $z > 0$. J^x and J^y are zero everywhere. Then, we get $F_Z = F_\lambda = 0$ with F_ψ given by,

$$F_\psi = \frac{2J^s_z\lambda}{1+\exp\left(\frac{2Z}{\lambda}\right)} \tag{31}$$

Now, we calculate the integrals with respect to the z co-ordinate for the terms in the main Lagrangian. We list down the equality among integrals,

$$\int dz\frac{-m_z}{\gamma}\dot{\phi} = \frac{-2Z}{\gamma}\dot{\psi} \tag{32}$$

$$\int dz\frac{A_{\text{ex}}}{\mu_0 M_s}(\partial_x\boldsymbol{m})^2 = \frac{2A_{\text{ex}}}{\mu_0 M_s\lambda} \tag{33}$$

$$\int dz\frac{H_\perp}{2}(\boldsymbol{m}.\hat{\boldsymbol{y}})^2 = H_\perp\lambda\sin^2\psi \tag{34}$$

$$\int dz\frac{-H_\parallel}{2}(\boldsymbol{m}.\hat{\boldsymbol{z}})^2 = H_\parallel(l-\lambda) \tag{35}$$

$$\int dz\frac{\alpha}{\gamma}\dot{\boldsymbol{m}}.\delta\boldsymbol{m} = \frac{2\alpha\lambda}{\gamma}\left(\frac{\dot{Z}\delta Z}{\lambda^2} + \dot{\psi}\delta\psi\right) + \frac{\alpha\pi^2}{6\gamma}\frac{\dot{\lambda}\delta\lambda}{\lambda} \tag{36}$$

$$\int dz(-\boldsymbol{m}.\boldsymbol{H}_{\text{ext}}) = -2H_{\text{ext}}Z \tag{37}$$

The final Lagrangian along with work is given by,

$$L = \mu_0 M_s wd\left[\frac{-2Z}{\gamma}\dot{\psi} - \frac{2A_{\text{ex}}}{\mu_0 M_s\lambda} - H_\perp\lambda\sin^2\psi + H_\parallel(l-\lambda) + \pi\lambda\left(H_x\cos\psi + H_y\sin\psi\right) + 2H_z Z\right] \tag{38}$$

$$\delta W = -\mu_0 M_s wd\left[\frac{2\alpha\lambda}{\gamma}\left(\frac{\dot{Z}.\delta Z}{\lambda^2} + \dot{\psi}.\delta\psi\right) + \frac{\alpha\pi^2}{6\gamma}\frac{\dot{\lambda}.\delta\lambda}{\lambda}\right] + F_Z\delta Z + F_\psi\delta\psi + F_\lambda\delta\lambda \tag{39}$$

The Euler-Lagrange equations then lead to,

$$-\dot{\psi}+\gamma H_{\text{ext}}=\frac{\alpha\dot{Z}}{\lambda} \tag{40}$$

$$\frac{\dot{Z}}{\lambda}-\gamma H_{\perp}\sin\psi\cos\psi=\alpha\dot{\psi}-\frac{\gamma\hbar J_z^s}{e\mu_0 M_s d}\frac{1}{1+\exp\left(\frac{2Z}{\lambda}\right)} \tag{41}$$

$$\frac{2A_{\text{ex}}}{\mu_0 M_s\lambda^2}-H_{\perp}\sin^2\psi-H_{\parallel}=\frac{\alpha\pi^2}{6\gamma}\frac{\dot{\lambda}}{\lambda} \tag{42}$$

Note that we have applied a field in $-z$ direction in the paper and hence in the notation of the paper, $H_{\text{ext}}=-H$. Similarly, the sign of J_z^s here is opposite of that in the paper.

So far, we have treated all coordinates $\{Z,\psi,\lambda\}$ on equal footing. However, λ is different from the other two in the sense that it changes the shape of Domain Wall instead of just coherent translation (Z) and rotation (ψ). Hence, under the validity of the rigid domain wall, the variation in λ should be small. This can be verified through numerical simulations as well. Using this approximation as a guide, we neglect the time derivative term (which is also proportional to α, thereby helping the approximation) in eq (42). Hence, we arrive at the equation of motion in $\{Z,\psi\}$ with λ as a function of ψ instead of an independent variable, given by $\lambda(\psi)=\sqrt{\dfrac{2A_{\text{ex}}}{\mu_0 M_s(H_{\parallel}+H_{\perp}\sin^2\psi)}}$.

3 Noise Analysis

At $T=0$, rigid domain wall approximation correspond to eqs (40) and (41) with qualifications about λ from the subsequent paragraph. At non-zero temperature, we have to add terms pertaining to noise in the aformentioned equations. In this section, we derive these terms and discuss the simulations of the stochastic differential equations. For analytical simplicity, we only consider the case when $H_{\perp}\ll H_{\parallel}$, such that λ can be taken to be a constant. Also, we consider the case of equilibrium without an external magnetic field or spin current and then assume that the noise sources remain the same in non-equilibrium. Under such circumstances, we can rewrite the final set of equations more generally by the following definitions,

$$p=\sqrt{\frac{2\mu_0 M_s wd}{\gamma}}\psi \tag{43}$$

$$x=\sqrt{\frac{2\mu_0 M_s wd}{\gamma}}Z \tag{44}$$

$$\mathscr{E}=\mu_0 M_s H_{\perp}wd\lambda\sin^2\psi \tag{45}$$

Note that with λ as a constant, the only term remaining in energy is the hard axis anisotropy. After some calculations, we can reduce eqs (40) and (41) in terms of x and p as,

$$\dot{x}=\frac{1}{1+\alpha^2}\left(\frac{\partial\mathscr{E}}{\partial p}-\alpha\lambda\frac{\partial\mathscr{E}}{\partial x}\right)+g_x N_x \tag{46}$$

$$\dot{p}=-\frac{1}{1+\alpha^2}\left(\frac{\partial\mathscr{E}}{\partial x}+\frac{\alpha}{\lambda}\frac{\partial\mathscr{E}}{\partial p}\right)+g_p N_p \tag{47}$$

where we have added two uncorrelated white source, N_x and N_p; g_x and g_p are assumed to be independent of Z and ψ. We assume $\langle N(t)N(t')\rangle=\dfrac{2\alpha k_B T}{1+\alpha^2}\delta(t-t')$ for both N_x and N_p. These assumptions are justified by deriving the expressions of g_x and g_p from the Fokker-Planck equations corresponding to the above Langevin equations. They are given as,

$$\frac{\partial P}{\partial t}=-\frac{\partial}{\partial x}\left[D_x^{(1)}P\right]-\frac{\partial}{\partial p}\left[D_p^{(1)}P\right]+\frac{\partial^2}{\partial x^2}\left[D_x^{(2)}P\right]+\frac{\partial^2}{\partial p^2}\left[D_p^{(2)}P\right] \tag{48}$$

where the drift and diffusion coefficients are given by,

$$D_x^{(1)}=\frac{1}{1+\alpha^2}\left(\frac{\partial\mathscr{E}}{\partial p}-\alpha\lambda\frac{\partial\mathscr{E}}{\partial x}\right)\qquad D_p^{(1)}=-\frac{1}{1+\alpha^2}\left(\frac{\partial\mathscr{E}}{\partial x}+\frac{\alpha}{\lambda}\frac{\partial\mathscr{E}}{\partial p}\right) \tag{49}$$

$$D_x^{(2)}=\alpha k_B T g_x^2\qquad D_p^{(2)}=\alpha k_B T g_p^2 \tag{50}$$

After a series of elementary manipulations, we can get the expressions for $g_x^2 = \lambda$ and $g_p^2 = 1/\lambda$ by demanding that $P = C\exp(-\mathscr{E}/k_BT)$ satisfies the Fokker-Planck equations in equilibrium, where C is some constant. Now we revert back to the original coordinates, $\{Z, \psi\}$. We define two noise terms, $N_Z = N_x - \alpha N_p$ and $N_\psi = \alpha N_x + N_p$ to simplify the final expression as,

$$\frac{\dot{Z}}{\lambda} = \gamma H_\perp \sin\psi\cos\psi + \alpha\dot{\psi} + \frac{\gamma\hbar J_z^s}{e\mu_0 M_s d}\frac{1}{1+\exp(2Z/\lambda)} + \sqrt{\frac{\gamma}{2\mu_0 M_s w d\lambda}}N_Z \tag{51}$$

$$\dot{\psi} = \gamma H_{\text{ext}} - \frac{\alpha\dot{Z}}{\lambda} + \sqrt{\frac{\gamma}{2\mu_0 M_s w d\lambda}}N_\psi \tag{52}$$

where we have added the external magnetic field and spin current back. Note that both N_Z and N_ψ are uncorrelated white noise sources with $\langle N(t)N(t')\rangle = 2\alpha k_B T\delta(t-t')$.

We then numerically simulated eqs (51) and (52) using Stratonovich calculus and second order Heunn scheme. We wrote the code in MATLAB by discretising time in units of $1ps$ and assuming the parameters as $M_s = 800kA/m$, $A_{\text{ex}} = 13pJ/m$, $H_\parallel = 32.9kA/m$, $H_\perp = 16.6kA/m$, $\gamma = 2.21\times10^5 m/(A-s)$ and $\alpha = 0.007$. To verify the code under equilibrium, we added a pinning potential of the form $0.5k_{\text{Pin}}Z^2$, with $k_{\text{Pin}} = 15.12\mu J/m^3$, and assumed a low temperature of $50K$ such that $\sin^2\psi \approx \psi^2$. Under such circumstances, the energy (refer eq (45)) becomes analogous to a particle in a harmonic potential and we verified that the equipartition theorem holds by finding the average value of Z^2 and ψ^2. All averages as well as any other statistical values were calculated after removing the initial motion for $40ns$ to allow the system to achieve time invariance.

We then applied the external inputs and assumed a room temperature of $300K$. We simulated the motion for $40\mu s$ and found the spectral function (again after removing the initial $40ns$ of motion). The simulation was run for various values of H_{ext} and J_z^s and we extracted the Q-factor in all the cases, which turned out to be between 500 and 1500. The simulation result for three cases is shown in the paper (see Fig 4 of the paper).

4 Micromagnetic Simulations

The theoretical analysis in the main paper was carried out using rigid domain wall approximation imposed on a 1 dimensional LLG with effective parameters for anisotropy. For a more quantitative treatment, we must take into account the dipolar interaction precisely instead of masking it with a local effective field as in eq (19). This, in general, might be a difficult problem to solve analytically. Hence, we simulate a magnetic domain wall under the conditions given in the paper to verify if the oscillations are happening and compare the motion with the one derived from theoretical analysis.

We consider a magnetic thin film of thickness $3nm$ (along y-axis), width $100nm$ (along x-axis) and length $800nm$ (along z-axis). Consider the LLG equation given in eq (1) with the effective magnetic field given by a sum of exchange field, crystalline anisotropy, field due to other dipoles and an external magnetic field.

$$\boldsymbol{H}_{\text{eff}} = \frac{2A_{\text{ex}}}{\mu_0 M_s}\partial_z^2\boldsymbol{m} + \frac{2K_a}{\mu_0 M_s}m_y\hat{\boldsymbol{y}} + \boldsymbol{H}_{\text{ext}} + \boldsymbol{H}_{\text{dip}} \tag{53}$$

where we assume crystalline anisotropy, K_a, to reduce the hard axis anisotropy due to demagnetisation field (this is required for rigid domain wall approximation to work); $\boldsymbol{H}_{\text{dip}}$ is the effective field due to the dipolar interaction between magnetizations. $\boldsymbol{H}_{\text{dip}}$ has been calculated as discussed by Miltat et al.[(2)]

We use finite difference method and fourth order Runge Kutta with a fixed time step of $0.2ps$ to simulate a tail-to-tail domain wall. As the thickness of the film is small compared to typical exchange length, we perform a 2-dimensional simulation with discretization in blocks of $4nm\times4nm$. The values of parameters are taken to be $A_{\text{ex}} = 13pJ/m$, $M_s = 800kA/m$, $K_a = 0.35MJ/m^3$ and $\alpha = 0.007$. To achieve the domain wall configuration, we simulate an infinite strip by adding additional magnetic charges at the two edges along the length[(3)]. These magnetic charges are due to the remaning left and right parts of the infinite strip, which are assumed to have a constant magnetization pointing along $\pm z$-axis. Under such conditions, we let the system equilibriate for a sufficient amount of time. After equilibrium, we observe that m_y is zero in the bulk and develop small non-zero values at the edges along the width. We average $\{m_x, m_y, m_z\}$ along the strip width (x-axis) to get the plots given in Fig 1. We have also shown the theoretical waveform derived from rigid domain wall approximation, $m_z(z) = \tanh\left(\frac{z-Z}{\lambda}\right)$ with the domain wall position, Z and width, λ chosen to fit the micromagnetic result. The system equilibriates at $Z = 64.2nm$ and $\lambda = 38.9nm$. We note that the initial location of the domain wall, Z, is dependent on the initial conditions of the simulations while the width, λ, is independent of it.

We then apply an external magnetic field of $8.75kA/m$ and vary the vertical spin current density to see the motion under these stimuli. The spin current applied is restricted to one half of the magnet as discussed in the paper. Fig 2 shows the motion

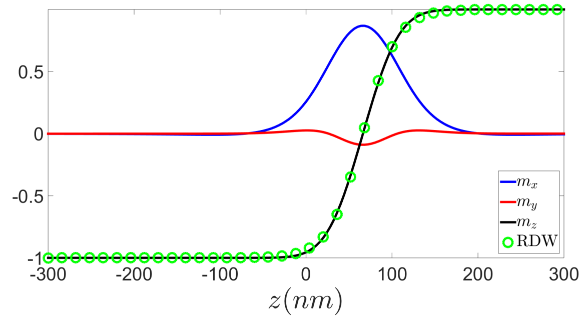

Figure 1. The equilibrium magnetization along the length of the magnet. Here $\{m_x, m_y, m_z\}$ denotes the average of the quantities taken along the width, x-axis. The dots ('RDW') refers to the fitted waveform of m_z in rigid domain wall approximation.

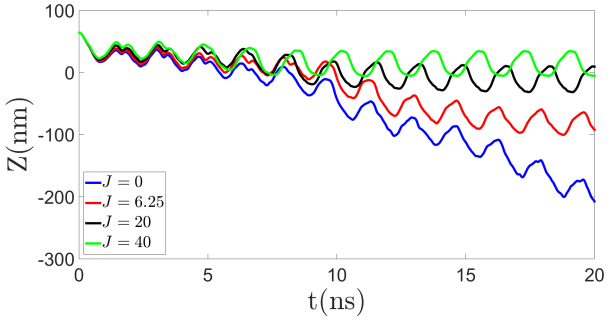

Figure 2. The motion of the domain wall under various values of spin current density with a magnetic field of $8.75kA/m$. The spin current is restricted to be in one half of the magnet as discussed in the paper.

under various values of spin current density, all in GA/m^2. The domain wall position has been defined as $(-l/2) * \langle m_z \rangle_x$ where the average is taken along x-direction. Note that we could as well define Z to be the point where $\langle m_z \rangle_x$ is zero. However, this distinction doesn't quantitatively affect the dynamics. At $J = 0$, we get back the usual oscillatory and drifting motion of the domain wall position. For non-zero small J, we still have a drifting domain wall but with a smaller velocity. For higher J, we get pure oscillations with the average domain wall position dependent on the spin current as discussed in the paper.

Now, we focus on the case of $J = 20GA/m^2$ where oscillations are stable. The oscillation frequency is $0.56GHz$ which agrees with the one derived from the rigid domain wall approximation in the paper, $0.61GHz$. As discussed in the paper, we have both translatory oscillations as governed by Z and rotation of the wall as governed by ψ. To show the rotation we plot the time evolution of $\langle m_y \rangle_x$ and $\langle m_x \rangle_x$ at the instantaneous domain wall position in Fig 3. We can see that they are oscillating with a phase difference of close to $\pi/2$. In rigid domain wall approximation, the oscillations of ψ occur with half the frequency of Z. This can be explicitly noted in micromagnetic simulations as well by plotting $\langle m_y \rangle_x$ at $z = Z(t)$ as a function of time and comparing it to $Z(t)$ itself as shown in Fig 4. From eq (41), we can see if the domain wall is sufficiently outside the region of non-zero spin current, $Z \gg \lambda$, the velocity of the domain wall is zero at $\psi = 0, \pm\pi/2, \pi$. Even though this condition is never achieved in the shown oscillations, it can be approximately noticed from the plot at the maxima of the domain wall position (where Z is still less than λ).

Fig 5 is a plot of $\langle m_z \rangle_x$ at the four points marked as $\{A, B, C, D\}$ in Fig 4. These waveforms can be fitted with rigid domain wall approximation with the domain wall position and width as fitting parameters. Using this, we can verify that at least for the averaged value of m_z, the rigid domain wall approximation is valid throughout the oscillations. This also illustrates the oscillations of the width of the domain wall.

To compare the micromagnetic simulations with theoretical analysis, we need to define $H_\perp$ and $H_\parallel$ through micromagnetics. The definition is given in terms of mean field of a mono domain magnet. We extract H_x as the mean field when all the magnetic moments are pointing along x axis. H_y and H_z are defined analogously. We then define $H_\parallel$ to be $H_x - H_z$ and $H_\perp$ as $H_y - H_x$. We note however that defining a particular $H_\perp$ and $H_\parallel$ can only give an estimate of the observables and cannot be used for quantitative correctness[4]. We get the values as $H_\parallel \approx 32.9kA/m$ and $H_\perp \approx 16.6kA/m$. Using these as effective parameters in the LLG equation, we calculate the amplitude of oscillations through the rigid domain wall approximation. We find that the

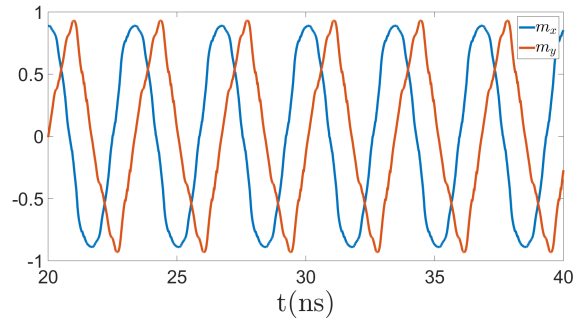

Figure 3. Depicting rotation in domain wall. Here m_y means the average value of m_y at the instantaneous position of the domain wall. The averaging is done across the width. m_x is computed similarly.

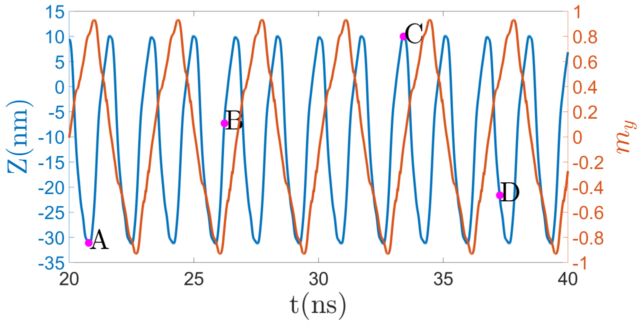

Figure 4. Illustrating the phase relation between the domain wall position and its rotation. Here again, m_y denotes the average value across the width at the instantaneous position of the domain wall.

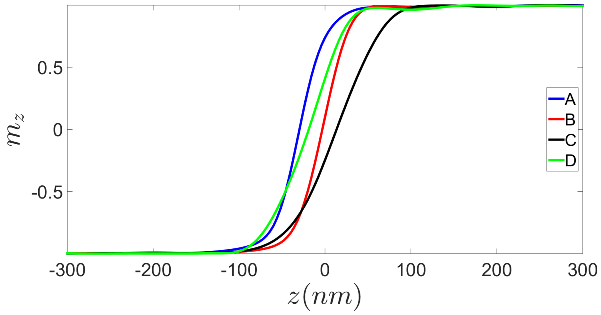

Figure 5. The plot of m_z averaged across the width for four different instances in the oscillations. This depicts the variation in width during different phases of the oscillations.

amplitude is of the same order as the micromagnetic prediction. The amplitude in micromagnetic simulations is approximately twice of the one derived from theoretical analysis in the paper. The factor of 2 could be because of a specific definition of $H_\perp$ and $H_\|$ and hence cannot be predicted easily. This is an evidence of the validity of the theoretical analysis carried out in the main paper.

References

1. Slonczewski, J. Current-driven excitation of magnetic multilayers. *Journal of Magnetism and Magnetic Materials* **159**, L1 – L7 (1996).

2. Miltat, J. E. & Donahue, M. J. *Numerical Micromagnetics: Finite Difference Methods* (John Wiley & Sons, Ltd, 2007). URL http://dx.doi.org/10.1002/9780470022184.hmm202.

3. McMichael, R. & Donahue, M. Head to head domain wall structures in thin magnetic strips. *Magnetics, IEEE Transactions on* **33**, 4167–4169 (1997).

4. Thiaville, A., Nakatani, Y., Miltat, J. & Vernier, N. Domain wall motion by spin-polarized current: a micromagnetic study. *Journal of Applied Physics* **95**, 7049–7051 (2004). URL http://scitation.aip.org/content/aip/journal/jap/95/11/10.1063/1.1667804.
