# Supplementary material for: Proposal for a Domain Wall Nano-Oscillator driven by Non-uniform Spin Currents
Source: Sci Rep. 2015 Sep 30;5:14647. doi: 10.1038/srep14647 (PMC4588506; doi:10.1038/srep14647)

Details of the Micromagnetic Simulations

Our micromagnetics code is written in MATLAB and based on finite difference method. It uses 4th order Runge-Kutta method to solve the LLG equations. The algorithm is based on following two references:

- A.J. Newell, W. Williams and D.J. Dunlop, Journal of Geophysical Research Vol. 98,No. B6, Pg 9551-9555, June 10, 1993.
- Numerical Micromagnetics : Finite Difference Methods, J. Miltat and M. Donahue, Handbook of Magnetism and Advanced Magnetic Materials, Edited by Helmut Kronmüller and Stuart Parkin. Volume 2: *Micromagnetism*

To validate the code, we compared our solution of standard problem 4a and 4b, given at http://www.ctcms.nist.gov/~rdm/mumag.org.html, with various other groups as illustrated in the following slides.

The parameters used are as follows:
Fixed time step=100 fs, Nx=128, Ny=32, a=3.90625 nm
Other paramters are the same as given for prob 4:
t=3 nm, γ0=2.211e6 m/A s , α=0.02, Ms=800 emu/cc, A_ex=1.3e-6 erg/cm

Comparison of Prob 4a

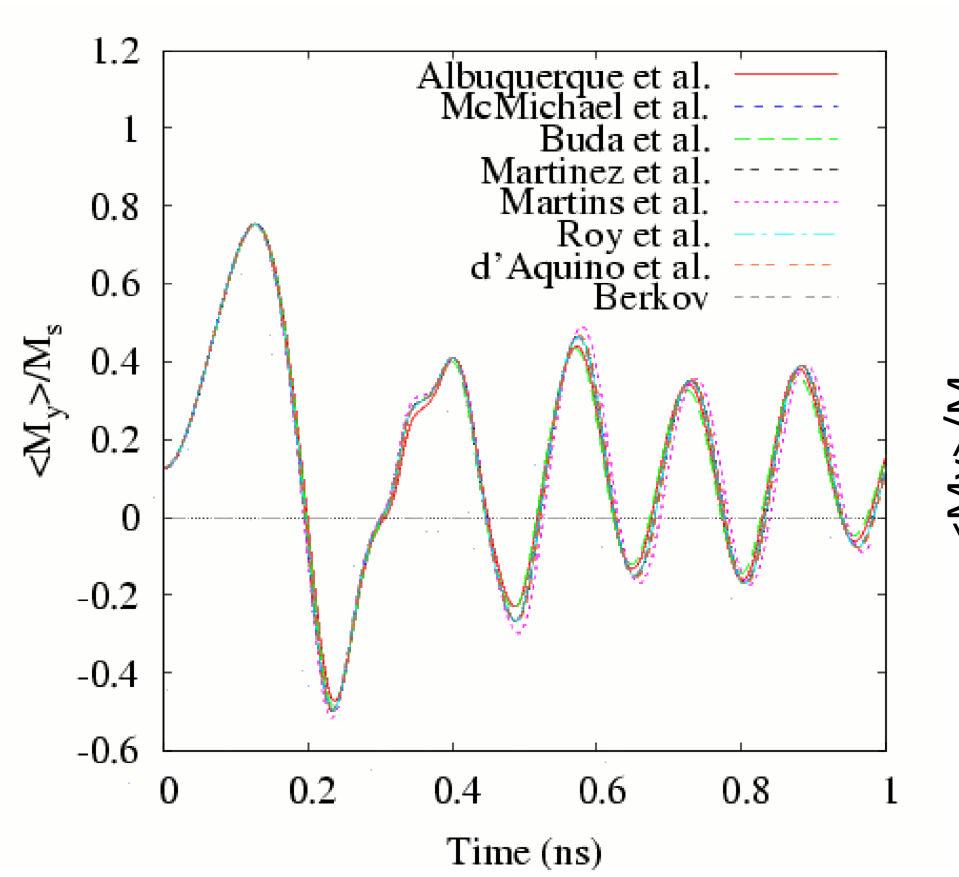

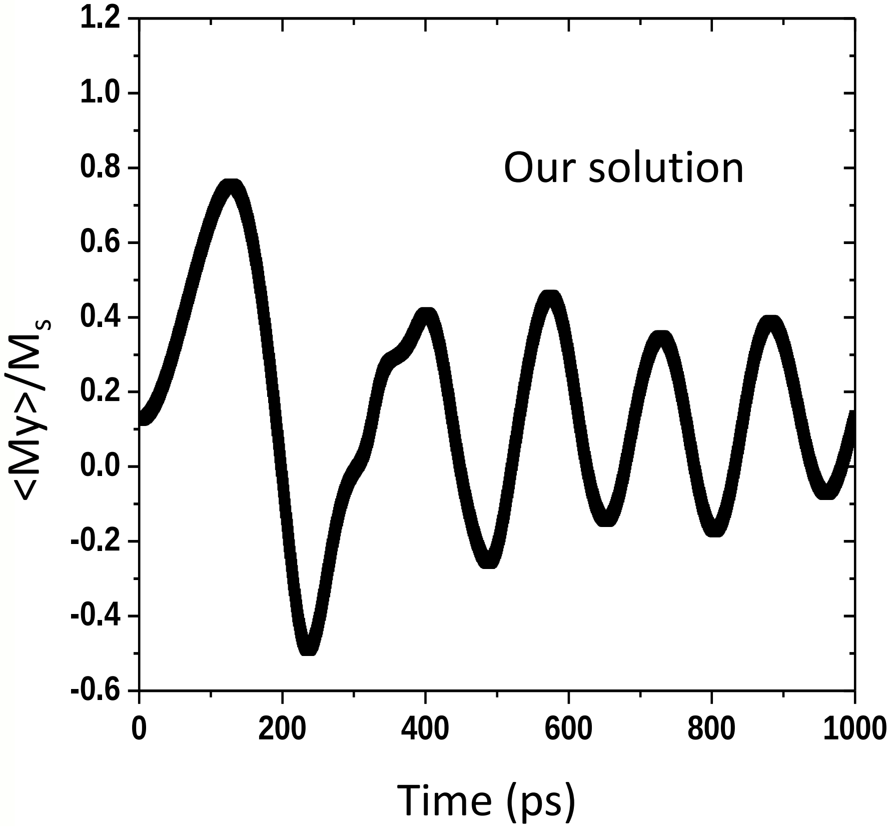

Comparison of Prob 4a

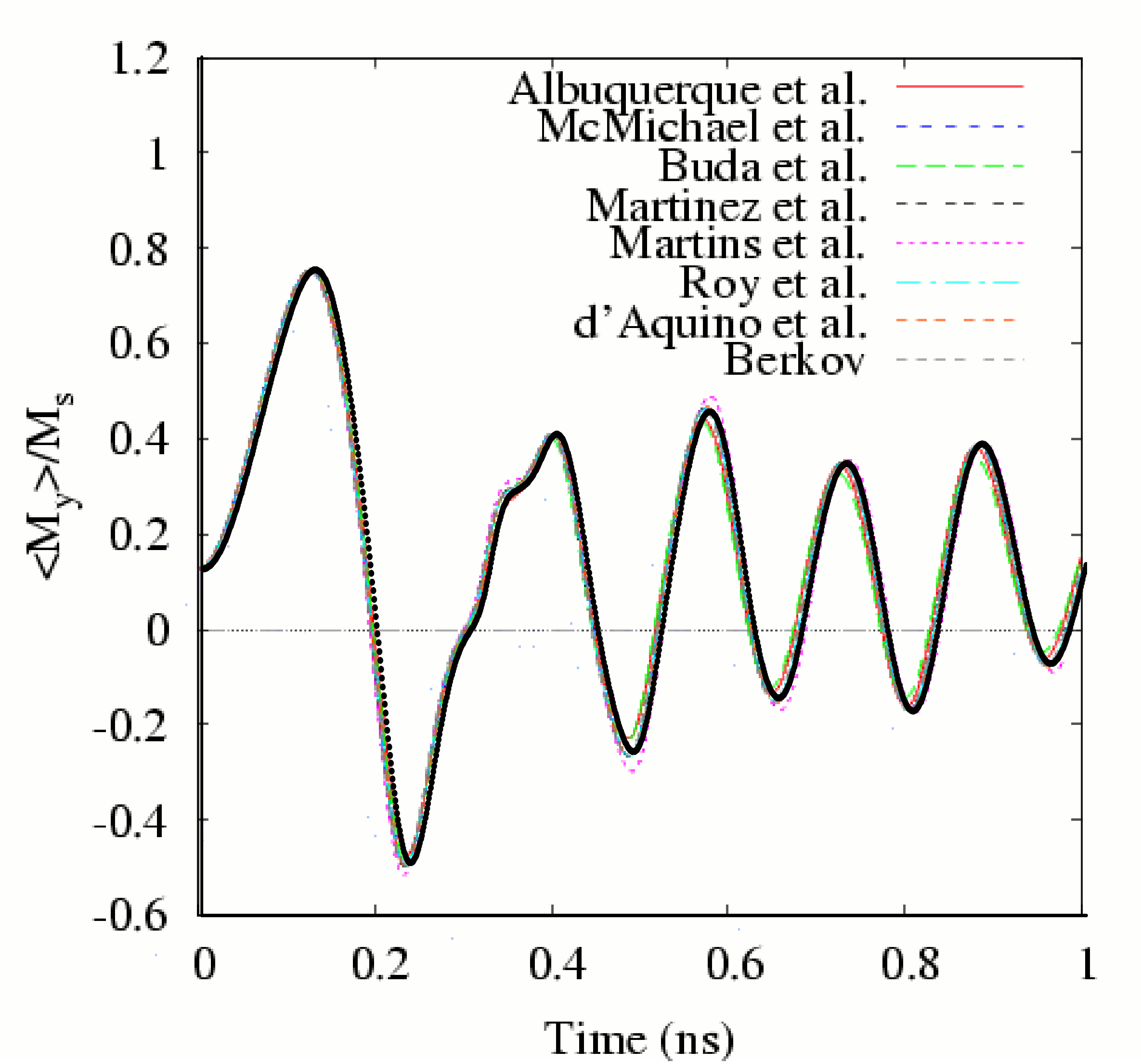

Comparison of Prob 4b

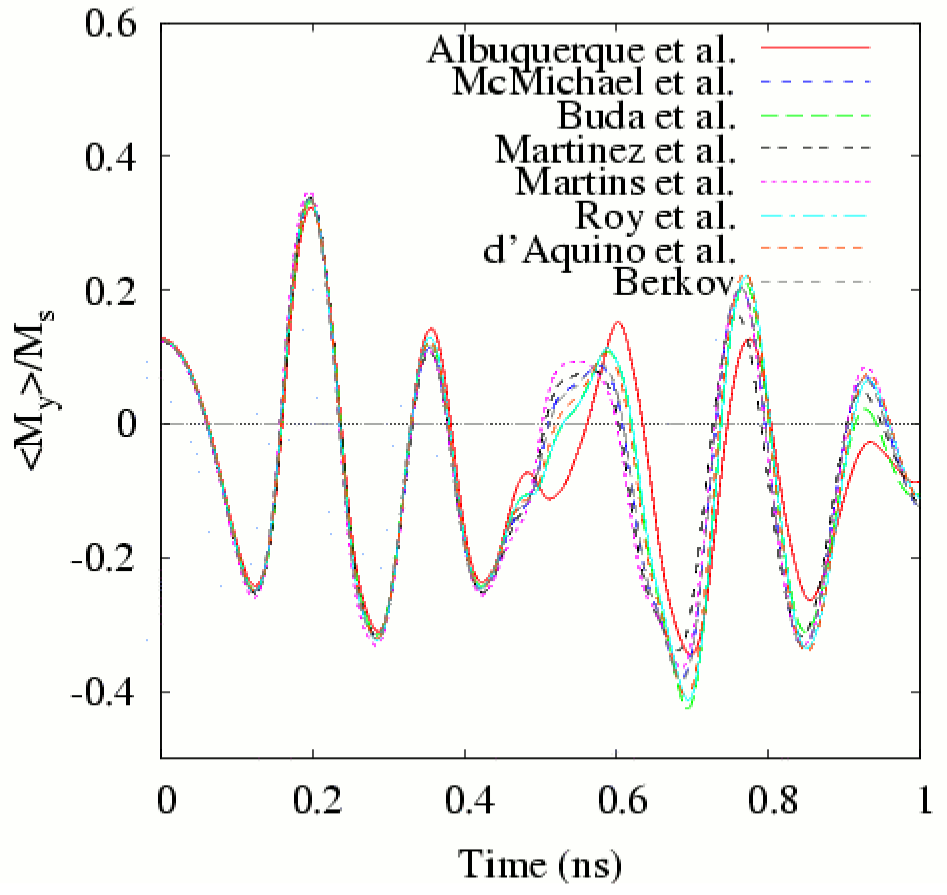

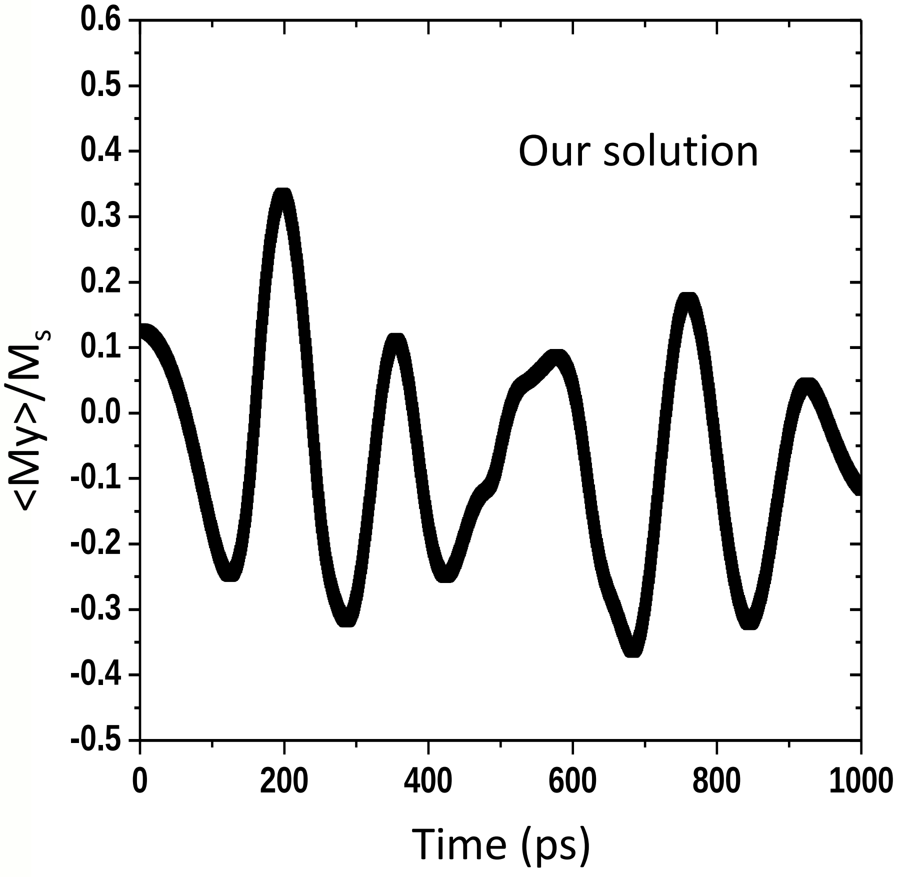

Comparison of Prob 4b

Our results match with the other groups for the initial simulation. The results of all the shown groups disagree after a certain time.

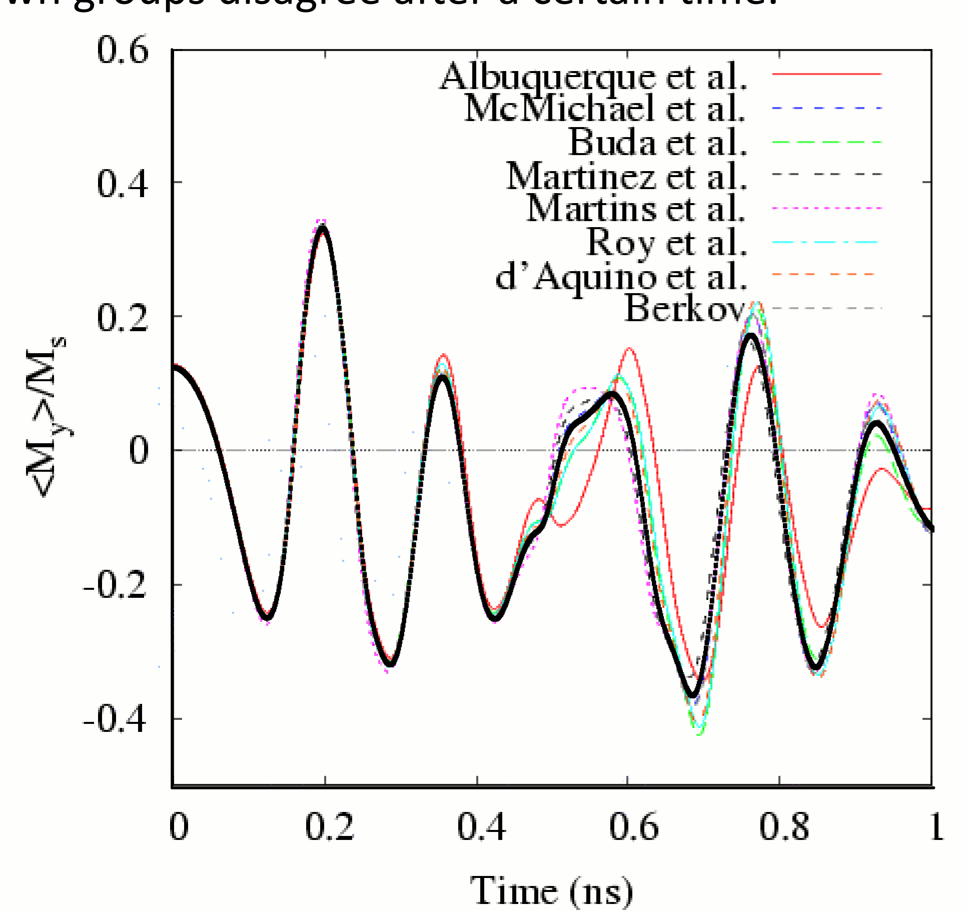

Comparison of Prob 4b

Zoom in of the simulation where the groups disagree

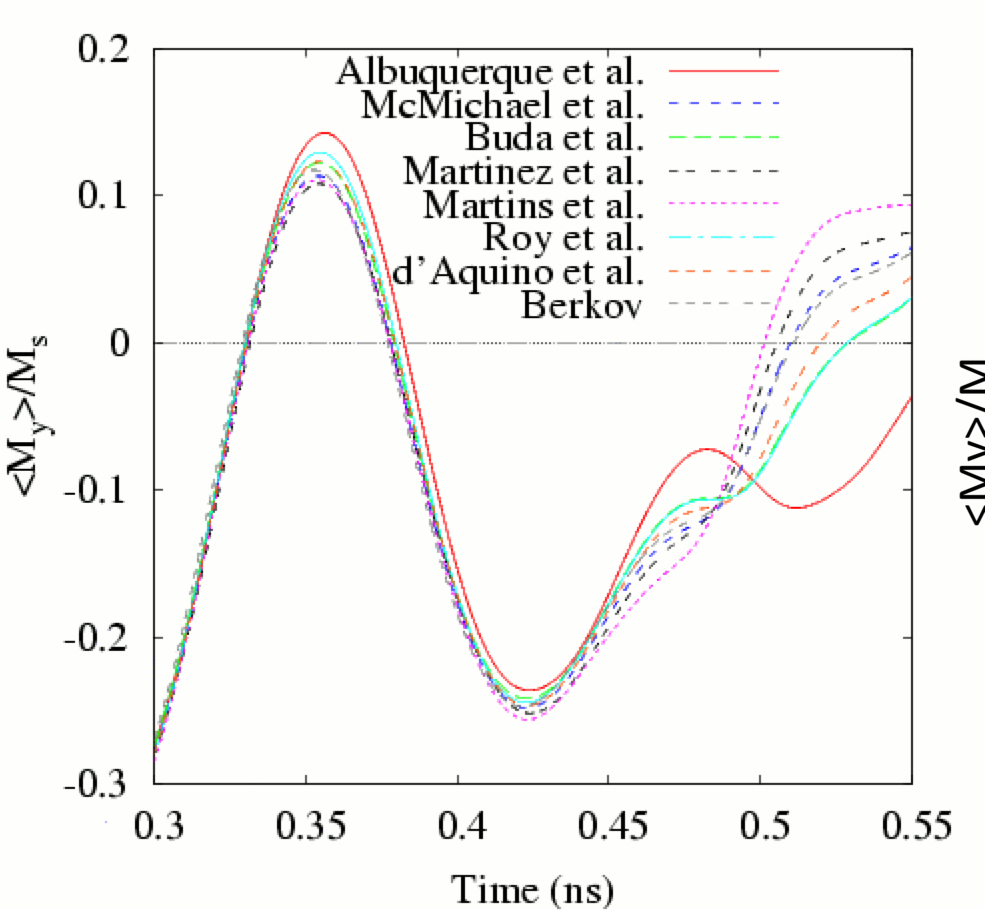

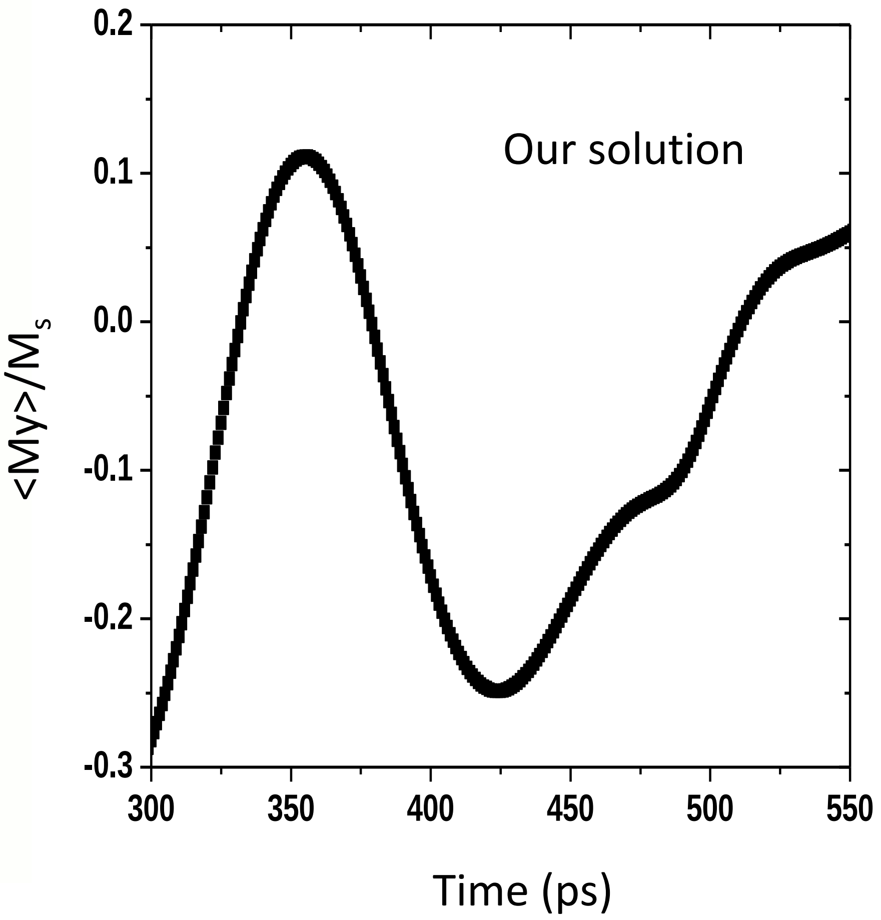

Comparison of Prob 4b

Zoom in of the simulation region where the groups disagree

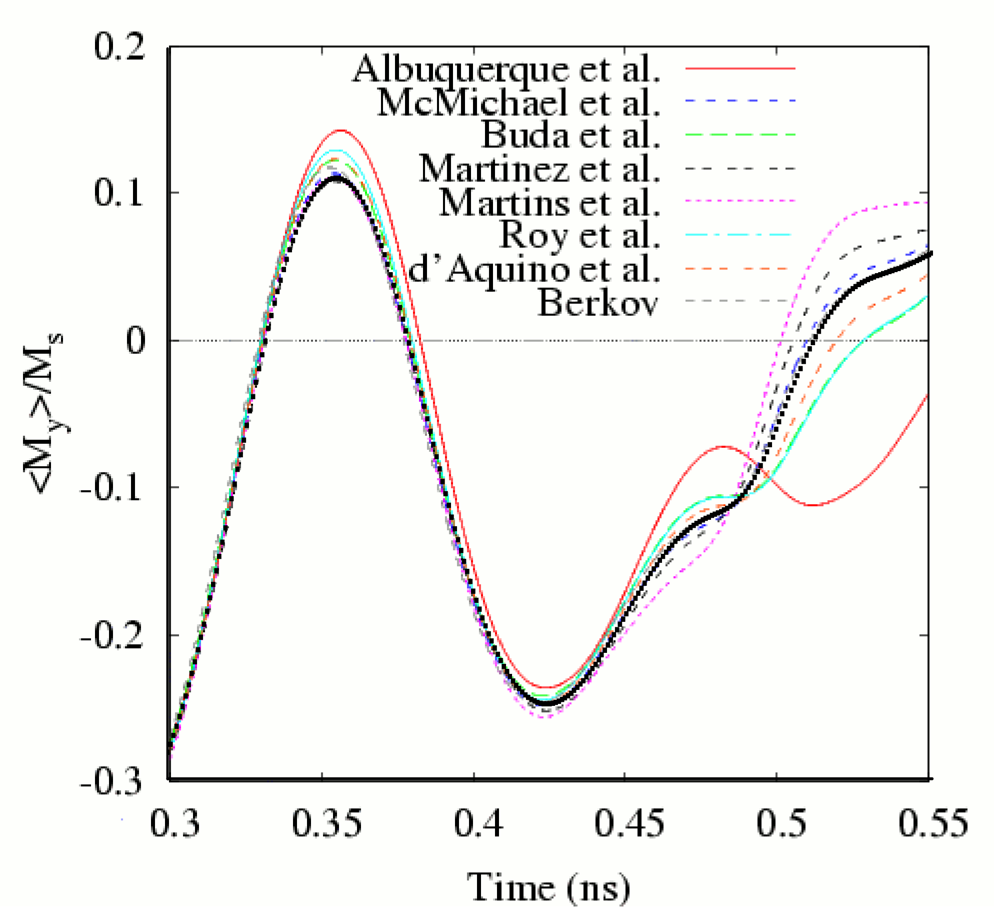

Supplement: Supplementary Information 1 [file srep14647-s2.pdf]
